# Role of Androgen Receptor in Melanoma: Mechanisms of Tumor Progression, Immune Evasion, and Therapeutic Implications

**DOI:** 10.3390/cancers17172828

**Published:** 2025-08-29

**Authors:** Claudia Lasalle, Yulu Wang, Maria T. Morales, Alessio Giubellino, Kyle T. Amber, Adrian P. Mansini

**Affiliations:** 1 Department of Dermatology, Rush University Medical Center, Chicago, IL 60612, USA; claudia_lasalle@rush.edu (C.L.); yulu_f_wang@rush.edu (Y.W.); mariateresa_morales@rush.edu (M.T.M.); kyle_amber@rush.edu (K.T.A.); 2 Department of Laboratory Medicine and Pathology, University of Minnesota, Minneapolis, MN 55455, USA; agiubell@umn.edu

**Keywords:** melanoma, androgen receptor, metastasis, immunosuppression, immunotherapy, chemotherapy, targeted therapy, androgen deprivation therapy

## Abstract

Melanoma is one of the most aggressive skin cancers, with increasing rates of occurrence. Although it has not traditionally been considered hormonally driven, recent evidence links androgen receptor (AR) signaling to important aspects of melanoma biology, including tumor growth, metastasis, immune evasion, and resistance to therapy. Mechanistically, AR promotes melanoma progression by activating a pro-metastatic gene program, suppressing anti-tumor immune responses, and altering the tumor microenvironment. Additionally, emerging data indicate AR’s involvement in resistance to chemotherapy and immune-based therapies. This review provides a comprehensive overview of AR’s intricate role in melanoma, focusing on its molecular mechanisms, its impact on immune evasion and therapy resistance, and its potential clinical applications. We also assess AR-targeted strategies, including androgen deprivation therapy and AR antagonists, to improve the effectiveness of chemotherapy, targeted therapy, and immunotherapy. Understanding AR’s role in melanoma could lead to new treatment options, particularly for sex-specific patient groups.

## Simple Summary

This review explains how the androgen receptor (AR), a protein typically associated with prostate cancer, also has a significant role in melanoma, one of the most aggressive skin cancers. **Metastasis (spread):** AR activates genes that increase the invasiveness of melanoma cells, aiding their movement, survival, and spread to other organs. **Immune evasion:** AR changes the tumor microenvironment, making it more difficult for immune cells to recognize and attack melanoma. It decreases antigen presentation, encourages immune-suppressive signals, and promotes “cold” tumors that are resistant to immunotherapy. **Therapy resistance:** AR signaling enables melanoma to evade targeted treatments such as BRAF/MEK inhibitors and diminishes the effectiveness of immune checkpoint blockade. Blocking AR can restore sensitivity in experimental models. **Sex differences:** Men, who generally have higher AR activity, often exhibit more aggressive melanoma and poorer outcomes compared to women. **Therapeutic potential:** New strategies to block or degrade AR (e.g., AR antagonists, selective degraders, PROTACs, RNA-based tools) are under investigation. Combining AR-targeted therapies with immunotherapy or other targeted treatments may enhance treatment outcomes. **Conclusions:** AR plays a crucial role in melanoma progression, immune suppression, and treatment resistance. Targeting AR presents a promising new therapeutic approach; however, further clinical studies are necessary to identify which patients would benefit most.

## 1. Introduction

Melanoma is a highly aggressive skin cancer with increasing global incidence and significant mortality, especially in metastatic cases [1]. Its incidence continues to grow worldwide, with men experiencing higher rates than women [2]. Geographic, ethnic, and socioeconomic factors, along with disparities in access to early detection and care, contribute to differences in incidence and outcomes [1]. While early-stage melanoma can often be successfully treated with surgical removal, managing unresectable or metastatic disease poses major challenges due to resistance to current treatments [3]. Conventional therapies like chemotherapy and radiotherapy frequently fail because of inherent or acquired resistance [4]. Immunotherapy, especially immune checkpoint inhibitors (ICIs), has greatly improved outcomes by leveraging the immune system to fight melanoma [5]. However, therapeutic resistance and immune evasion still present significant obstacles to long-term success. Therefore, new molecular targets are urgently needed to enhance treatment responses and survival rates in patients with advanced disease.

The androgen receptor (AR) is a ligand-activated transcription factor that belongs to the nuclear receptor superfamily and is a key mediator of androgen signaling [6]. AR plays a crucial role in regulating gene expression, DNA repair, and maintaining genome integrity [7,8]. In prostate cancer, AR signaling is a major driver of tumorigenesis by regulating genes involved in proliferation, survival, and differentiation [9,10]. Consequently, androgen deprivation therapy is a mainstay of treatment [11]. While traditionally overlooked in non-hormone-dependent cancers, AR is now gaining attention for its potential role in malignancies such as melanoma [7,12,13,14,15].

Epidemiological data demonstrate sex-based disparities in melanoma incidence and outcomes, with men exhibiting higher incidence and mortality rates than women [16,17,18,19,20]. This phenomenon is not entirely explained by behavioral or environmental factors [15,21]. These observations have sparked interest in the influence of sex hormones and their receptors, including AR, on melanoma biology.

In this review, we explore the multifaceted role of AR in melanoma, with a focus on three major domains: metastasis, immunosuppression, and therapeutic resistance. We also discuss the integration of AR-targeted strategies with existing treatment modalities, highlighting emerging approaches and the potential for sex-specific interventions.

## 2. Androgen Receptor in Melanoma Metastasis

Metastasis remains the leading cause of melanoma-related mortality and represents a major obstacle to effective treatment [22]. BRAF and NRAS oncogenic mutations, as well as environmental factors such as ultraviolet (UV) exposure, have traditionally dominated discussions of melanoma progression [23,24]. Recent data implicate sex hormones, particularly the AR, in modulating the metastatic behavior of melanoma cells.

AR is present at low levels in all primary melanocytes, but to varying degrees among melanoma cells. Some tumors have high levels of AR protein and mRNA (AR^+^), while others show minimal to no expression (AR^−^) [14,15,25]. When expressed at high levels, AR is mainly localized to the nucleus of melanoma cells, whereas it is predominantly distributed perinuclearly in low AR-expressing melanoma cell lines and primary melanocytes [25].

AR promotes melanoma invasiveness through multiple integrated pathways. Wang et al. demonstrated that AR enhances the expression of miR-539-3p, a microRNA that suppresses the deubiquitinase USP13, leading to destabilization of MITF, a melanocytic lineage transcription factor critical for maintaining cell differentiation [14]. The subsequent downregulation of MITF favors the expression of AXL, a receptor tyrosine kinase associated with invasive and therapy-resistant phenotypes, thereby promoting epithelial-to-mesenchymal transition (EMT) and metastatic potential. Furthermore, utilizing an in vivo melanoma metastasis model, they demonstrated that AR increases melanoma lung metastasis by modifying MITF [14].

Supporting evidence from prostate cancer shows that AR can upregulate EMT-related transcription factors, including Twist-related protein 1 (TWIST1) and zinc finger protein SNAI1 (SNAI1). Furthermore, AR upregulates matrix metalloproteinases (MMPs) such as MMP2 and MMP9, which facilitate matrix degradation [26,27,28,29,30].

Although these mechanisms are best defined in prostate cancer, in vivo melanoma models confirm that high AR activity enhances metastatic spread [25].

Ma et al., working with several melanoma cell lines and primary melanoma cells, demonstrated that silencing AR in melanoma cells leads to the downregulation of *CDCA7L*, thereby inducing cellular senescence [25]. Notably, *CDCA7L* is a transcriptional repressor that interacts with C-MYC, sharing oncogenic function. They also observed the upregulation of mRNA for *CDKN1A* and several immunomodulators, such as intercellular adhesion molecule 1 (*ICAM1*) and the pro-inflammatory cytokine interleukin 6 (*IL-6*). Furthermore, several genes involved in melanogenesis, differentiation, and melanoma progression were upregulated or downregulated following AR silencing. These data support the functional role of AR in melanoma development and metastasis.

Recent work by Liu et al. identified the exact mechanism by which AR controls the transcriptional increase in fucosyltransferase 4 (FUT4) expression, highlighting its key role in enhancing melanoma invasiveness. In this process, AR-driven FUT4 signaling alters cell–cell adhesion by disrupting N-cadherin-catenin-based junctional complexes between melanoma cells, which impacts adherens junctions (AJs) and promotes cellular spread [15]. Additionally, they demonstrated that androgen stimulation in melanoma cells did not significantly affect genes typically regulated by AR in prostate cancer, indicating that AR drives a distinct transcriptional program in melanoma cells.

Although AR has been implicated in promoting melanoma aggressiveness and metastasis, clinical data suggest its role may be highly context-dependent. Singh et al., analyzing the Cancer Genome Atlas human cutaneous melanoma (TCGA SKCM) dataset (353 patients with RNA data), reported that high AR protein levels correlated with improved overall survival, particularly in female patients and in RAS-mutant subtypes, whereas this association was not observed in BRAF, NF1, or triple-wild-type melanomas [31]. Notably, the survival benefit was lost when ulceration, a strong prognostic factor, was included in the analysis, underscoring the complexity of AR biology. Several mechanisms could account for these divergent observations, including isoform-specific signaling, post-translational modifications, or compensatory immune activation. In prostate cancer, AR splice variants lacking the ligand-binding domain are well recognized and drive therapy resistance, but the existence and function of AR isoforms in melanoma remain unexplored. Thus, AR may exert either tumor-promoting or tumor-suppressive effects depending on the molecular and clinical context, highlighting the need for further mechanistic studies. The sex-based disparities in melanoma outcomes further support the involvement of AR in disease progression. Male patients tend to exhibit higher AR activity and worse clinical outcomes [32], even when accounting for environmental and behavioral factors, indicating that androgen signaling can contribute to the more aggressive disease observed in men.

Further research is needed to clarify how AR expression and activity influence metastasis and treatment response. Future studies should investigate how AR signaling intersects with other oncogenic and immune pathways, and how hormonal modulation can be integrated into therapeutic strategies. These efforts can uncover new opportunities for precision medicine approaches in melanoma, including AR-targeted interventions for select patient populations.

## 3. Androgen Receptor-Mediated Immune Evasion and Tumor Microenvironment Reprogramming

AR is expressed by nearly all immune cells, and its activation affects both innate and adaptive immunity [8,33]. In immune cells, AR signaling has dual effects. While in neutrophils, AR activation stimulates pro-inflammatory cytokines, such as TNF-α, by regulating promoter activity [34], in macrophages, it controls their polarization and differentiation toward the pro-tumor M2 phenotype [35].

In peripheral T cells, AR activation inhibits proliferation by limiting IL-2 signaling. It also reduces the differentiation of Th1 cells from naïve precursors and decreases IL-12 expression through inhibition of STAT4 activation [33,36]. Furthermore, AR signaling suppresses IFN-γ production in T cells by repressing lfng transcription. In B cells, AR activity inhibits lymphopoiesis [37].

Recent studies have implicated AR signaling as a critical regulator of the tumor microenvironment (TME) in melanoma. TME plays a pivotal role in melanoma progression and therapy resistance, particularly by modulating the immune landscape [38,39].

Studies based on the analysis of AR expression and activity using TCGA SKCM data show that AR expression in tumors correlates with exhausted CD8+ T cells [40]. Increased AR activity is also negatively associated with immune cell infiltration in patients with melanoma [41]. In fact, patients with low levels of AR activity show improved response to ICB.

AR correlates with the upregulation of immune checkpoint molecules. While AR directly regulates B7-H3 in prostate cancer, its role in melanoma is less defined; nonetheless, B7-H3 is highly expressed and contributes to immune evasion [42,43,44]. B7-H3 is a transmembrane glycoprotein implicated in suppressing anti-tumor immune responses and promoting immune tolerance [45,46]. In melanoma, its overexpression is associated with an immunosuppressive TME, characterized by reduced infiltration of CD8^+^ T lymphocytes and increased extracellular matrix collagen deposition. This profile defines the so-called “armored-cold” phenotype, characterized by high stromal density, immune exclusion, and a poor response to checkpoint inhibitors [42]. Our group is currently exploring this potential regulatory axis.

Melanoma cells release soluble ligands such as the major histocompatibility complex class I chain-related protein A (MICA) and B (MICB). These bind to the activating receptor NKG2D on NK cells, γδ T cells, and CD8+ T cells [47,48]. The interaction triggers receptor internalization and degradation, leading to immunosuppression [49]. AR activation promotes MICA shedding through the upregulation of ADAM10 and the formation of an AR/ADAM10/β1-integrin complex [28,39]. This process abrogates immune cell activation and cytotoxicity.

A subset of melanoma cells displays diminished antigen-presenting capacity, including the downregulation of major histocompatibility complex (MHC) class I and II molecules and essential proteins required for the adequate functioning of the MHC, such as the transporter associated with antigen processing protein (TAP) [41,50,51,52]. Studies show significantly lower TAP1/2 expression in melanoma cells compared to melanocytes, with a corresponding decrease in HLA class I expression [52]. These changes impair the recognition of cytotoxic T lymphocytes and hinder immune-mediated tumor clearance. This immune evasion may be driven, at least in part, by AR-mediated repression or shedding of MHCI pathway components [15,48].

Activation of the AR in immune cells has been linked to immunosuppression by altering the function and recruitment of various immune cell subsets, including T cells, B cells, and macrophages, within the TME [30,33]. In some cancers, such as prostate and breast cancers, elevated AR activity is associated with increased accumulation of T regulatory cells (Tregs) and myeloid-derived suppressor cells (MDSCs) [53,54]. However, little is known about AR expression and its effects on immune cell recruitment in melanoma.

Ma et al. silenced AR in three melanoma cell lines and identified a 155-gene signature linked to AR loss. They then compared this signature with gene expression data from 469 cutaneous melanomas in the TCGA SKCM cohort. Tumors resembling the AR-silencing signature had higher infiltration of B cells, CD4^+^ and CD8^+^ T cells, and macrophages [25]. These tumors were also enriched for pro-inflammatory M1-like macrophages, rather than immunosuppressive M2 subsets, and showed increased CD4^+^ memory T cells [25]. These data indicate that AR actively represses anti-tumor immune infiltration and promotes a tolerogenic microenvironment (an immune-suppressive state where the tumor educates immune cells to tolerate its presence rather than attack it).

**Figure 1:**
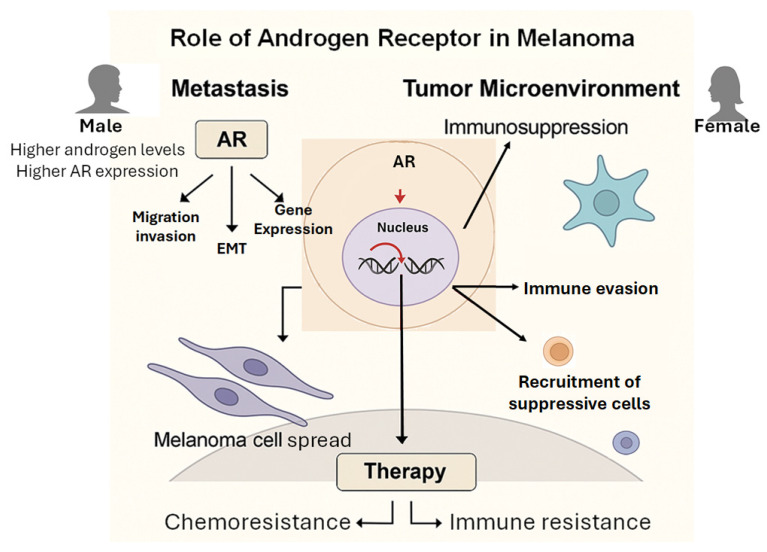
Mechanisms of AR-mediated melanoma progression: metastasis and immune evasion. This schematic illustrates the complex role of AR signaling in promoting melanoma metastasis and shaping the immunosuppressive tumor microenvironment (TME). AR resides in the cytoplasm and, upon activation, translocates to the nucleus to regulate target gene expression (red arrows). AR activation in melanoma cells stimulates the epithelial–mesenchymal transition (EMT) by lowering MITF levels and increasing AXL and FUT4, thereby enhancing the invasiveness and metastatic potential of these cells. AR also supports immune evasion by reducing antigen presentation (by repressing TAP1/2 and MHC-I) and promoting MICA shedding through ADAM10. Additionally, AR signaling modifies the TME toward a tolerogenic state by decreasing infiltration of cytotoxic T and B cells, encouraging M2 macrophage polarization, and increasing the presence of Tregs and exhausted T cells.

A schematic overview of AR-mediated mechanisms in melanoma metastasis and immunosuppression is shown in Figure 1.

## 4. Androgen Receptor and Therapy Resistance

Resistance to targeted and immune-based therapies presents a significant clinical challenge in melanoma treatment. Multiple studies emphasize AR as a key factor in the development of resistance.

AR upregulation is a key adaptive mechanism in BRAF-mutant melanoma. Elevated AR activity sustains MAPK signaling even in the presence of BRAF and MEK inhibitors. It also drives resistance through activation of TGFβ and EGFR signaling. Furthermore, AR stimulates SERPINE1 expression, which enhances motility and metastasis. Clinical data confirm strong correlations between AR and SERPINE1 expression, particularly in metastatic lesions [7,55].

Targeting AR reverses therapeutic resistance in preclinical models. AR inhibitors, such as AZD3514, ARCC4, and enzalutamide, decrease EGFR and SERPINE1 expression and reduce tumorigenicity in BRAF inhibitor-resistant melanoma cells [7]. Pharmacologic AR blockade makes tumors more sensitive to BRAF/MEK inhibitors and improves regression in vivo, in both male and female models [13].

AR also plays a critical role in resistance to immunotherapy. Androgen deprivation therapy (ADT) enhances the response to PD-1 blockade in murine melanoma models by increasing tumor-infiltrating lymphocytes and reinvigorating cytotoxic T cell activity [48,56,57,58]. These findings suggest that AR antagonism may synergize with ICIs, supporting the development of combinatorial therapeutic strategies.

Notably, sex-based differences in immunotherapy outcomes may also reflect differential AR signaling. Sex hormones influence the expression and function of checkpoint proteins such as PD-1 and PD-L1. Studies in melanoma models show that female mice respond more effectively than males to anti-PD-L1 therapy [59], partly due to a greater reduction in Treg activity. Higher systemic androgen levels in males may enhance AR-driven immunosuppression, limiting immunotherapy efficacy, whereas lower androgen levels in females may favor more robust immune responses [60].

However, clinical data are mixed. A pooled analysis of multiple trials reported that men with advanced melanoma demonstrated better immunotherapy responses than women, both short- and long-term, with higher peripheral immune activation observed in male patients [61]. Although most mechanistic data on AR and immune suppression come from prostate cancer and other androgen-sensitive tumors, emerging studies in melanoma suggest a similar paradigm. This highlights the importance of sex-specific tumor biology in shaping immune responses and treatment outcomes.

Therapeutically, several AR-targeting strategies are under investigation. However, the systemic nature of ADT and its associated side effects, including metabolic disturbances, cardiovascular risk, and osteoporosis [62,63,64,65], limit its long-term applicability outside of prostate cancer. More selective pharmacologic interventions, including AR antagonists like enzalutamide, apalutamide, and darolutamide, directly block AR transcriptional activity [64] and have shown efficacy in metastatic prostate cancer, though their use in melanoma remains investigational [15,66,67].

A summary of the AR-driven mechanisms and their implications in melanoma is provided in Table 1.

While preclinical models consistently demonstrate the benefits of AR inhibition, restoring sensitivity to BRAF/MEK inhibitors and enhancing the efficacy of immunotherapy, clinical evidence remains sparse. Few melanoma-focused clinical trials have tested AR inhibitors, and most are small early-phase studies. Challenges include patient stratification based on AR expression, limited biomarker validation, and concerns regarding systemic toxicity. Addressing these translational barriers will be essential for advancing AR-targeted strategies in melanoma.

## 5. Clinical Translation and Future Directions

Translational efforts to AR-targeted therapies in melanoma treatment are gaining momentum. For example, in a recent study, it was shown that patients with melanoma with high AR activity and treated with ICIs respond worse to the therapy compared with those patients with low AR activity [41]. Furthermore, an early-phase clinical trial investigating the use of bicalutamide, an AR antagonist, in combination with nivolumab (PD-1 checkpoint inhibitor), has shown encouraging signals, particularly in patients with AR-positive tumors [66]. This trial indicates that inhibiting AR can synergize with immunotherapy, leading to improved outcomes for subsets of melanoma patients who have shown resistance to conventional treatments.

Beyond AR antagonism, novel agents such as selective AR degraders (SARDs, drugs that break down AR proteins) and proteolysis-targeting chimeras (PROTACs, engineered molecules that tag AR for destruction by the cell’s protein recycling machinery) offer the potential for more comprehensive AR inhibition by receptor degradation [68]. Early preclinical data indicate that PROTAC-mediated AR degradation not only reduces tumor growth but also enhances anti-tumor immunity [68,69]. Indeed, ARCC4 showed therapeutic effects in melanoma models [7].

Targeting AR can also potentiate the efficacy of emerging therapies such as adoptive cell transfer and oncolytic virotherapy. Given AR’s role in modulating immune cell recruitment and function, its inhibition might reprogram the TME to favor these immune-based modalities.

Innovative approaches, including RNA interference (RNAi) strategies that silence AR expression, have shown potent anti-tumor effects in vitro and in vivo [70]. Nanoparticle-based delivery systems may further improve the specificity and clinical feasibility of AR-directed RNA therapeutics.

On the other hand, as with other targeted therapies, prolonged AR inhibition may lead to adaptive resistance via compensatory signaling, genetic alterations, or microenvironmental reprogramming. Therefore, biomarker-driven approaches will be essential to maximize clinical benefit.

An additional future perspective is the potential use of Selective Androgen Receptor Modulators (SARMs) in treating melanoma [71]. Although speculative, SARMs may represent a new class of agents that selectively inhibit AR signaling in tumor tissue while limiting systemic toxicity. Tissue specificity is central to their design. Depending on context, SARMs can act as agonists or antagonists. In muscle and bone, they often behave as agonists, promoting anabolic effects. In contrast, in prostate and skin, some SARMs act as antagonists or partial agonists. Their activity depends on chemical structure, AR co-regulators, and chromatin accessibility [72,73]. This tissue-specific modulation has been well described for agents such as enobosarm (GTx-024) and LGD-4033 (ligandrol), which show anabolic activity with limited effects on prostate tissue in preclinical and early-phase clinical studies [74,75].

In melanoma, gene silencing or pharmacological inhibition of AR in melanoma cell lines suppresses proliferation and induces cellular senescence [25]. Additionally, AR silencing causes chromosomal DNA breakage and the release of double-strand DNA into the cytosol, triggering STING expression. Mechanistically, AR silencing disassembles Ku70/Ku80 DNA repair proteins from the RNA Pol II complex, increasing DNA damage at transcription sites [25]. Based on these insights, SARMs with antagonistic properties in melanoma tissue might be developed to block AR signaling without the systemic endocrine side effects associated with ADT or full AR antagonists such as enzalutamide [76].

The side effects of AR-targeted therapies warrant particular attention in melanoma. While ADT is standard in prostate cancer, its use in patients without prostatic malignancy raises safety concerns. Adverse events such as metabolic syndrome, cardiovascular complications, osteoporosis, and fatigue can significantly impact quality of life, especially in patients with pre-existing comorbidities. A critical evaluation of risk–benefit ratios, alongside the development of more selective tissue-targeted AR inhibitors, will be required before the widespread clinical use of AR inhibitors in melanoma can be justified.

Nevertheless, direct preclinical or clinical evidence for SARMs in melanoma is currently lacking. Potential strategies could include engineering SARMs with cutaneous-selective AR antagonism or combining SARMs with immunotherapies to enhance T cell infiltration, reduce immune checkpoint expression, and improve antigen presentation within the TME. However, caution is warranted. SARMs are not currently approved for cancer treatment, and their effects in melanoma have not been systematically studied. Furthermore, in AR+ tumors, some SARMs can act as partial agonists or even stimulate AR activity depending on the TME, co-regulator landscape, and ligand concentration, potentially promoting tumor progression [72,77].

**Figure 2:**
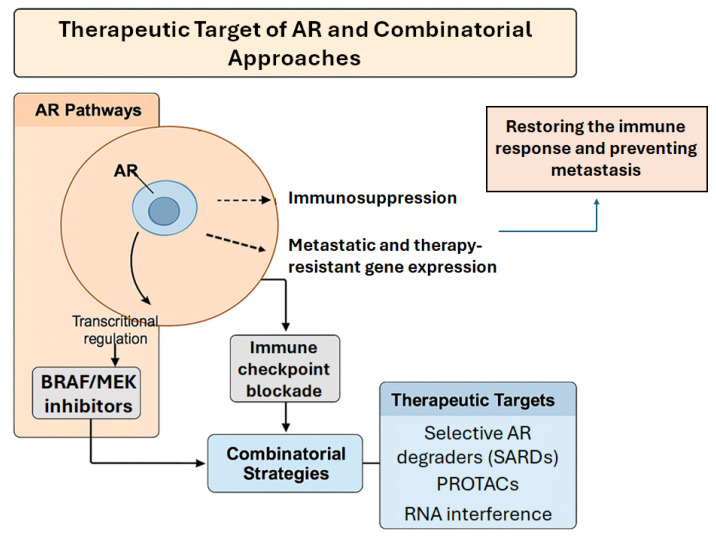
Therapeutic targeting of AR signaling in melanoma: strategies and combinations. This diagram illustrates how AR signaling promotes melanoma progression by inducing immunosuppression and expression of premetastatic and therapy-resistant genes. Targeted therapies, including selective AR degraders (SARDs), PROTACs, and RNA interference, can restore immune surveillance, boost MHC-I expression, and suppress metastatic programs. Combining AR-targeted treatments with immune checkpoint blockade or kinase inhibitors offers a promising approach to overcoming resistance and enhancing patient outcomes in AR-positive melanoma.

While some AR+ tumors with high AR activity respond well to ADT or AR antagonists, others exhibit intrinsic resistance, likely due to the heterogeneity of AR signaling and its complex interactions with other oncogenic pathways. This variability highlights the importance of precision medicine approaches that consider not only AR expression but also additional biomarkers, such as tumor mutational burden, immune checkpoint expression, and other factors that influence tumor behavior. Stratifying patients based on these criteria might enhance the therapeutic efficacy of AR-targeted therapies and help overcome the challenge of resistance by combining different therapeutic approaches (Figure 2).

An additional consideration in the clinical application of AR-targeted therapies is the potential for sex-based differences in AR signaling. Epidemiological studies consistently indicate that melanoma is more aggressive and associated with a worse prognosis in men [20], which may be influenced by differential AR signaling. Tailoring AR-targeted therapies to account for these sex-specific differences might provide a therapeutic advantage, addressing the unique challenges faced by male and female patients. Gender-specific treatment strategies might optimize the efficacy of AR inhibitors, reducing the likelihood of resistance and enhancing overall patient outcomes.

## 6. Conclusions

The androgen receptor is a critical regulator of melanoma aggressiveness, influencing metastasis, immune evasion, and therapy resistance.

Mechanistic studies reveal that AR orchestrates a pro-tumorigenic program through transcriptional control of EMT-related genes, suppression of antigen presentation, induction of immunosuppressive cytokines, and persistence of survival signaling. These findings not only highlight AR as a biomarker of disease progression but also establish it as a viable therapeutic target.

The development of AR antagonists, degraders, and RNA-based inhibitors opens the door for integrated treatment approaches that combine AR-targeted therapies with existing immunotherapies and kinase inhibitors. Furthermore, acknowledging sex-specific differences in AR signaling provides an opportunity to optimize treatment strategies and personalize care.

Future research should focus on refining biomarkers of AR activity, stratifying patients based on AR expression and downstream signaling signatures, and designing clinical trials that evaluate the efficacy of AR-targeted agents across diverse patient populations. A comprehensive understanding of AR’s role in melanoma will enable the translation of these insights into tangible clinical benefit.

The integration of AR inhibitors into the therapeutic landscape for melanoma presents a promising strategy to complement existing treatments and potentially overcome resistance to chemotherapy, targeted therapy, and immunotherapy. Ongoing clinical trials will be pivotal in defining the optimal combinations, sequencing, and patient selection criteria for these therapies. Thus, further studies are needed to refine our understanding of how AR interacts with melanoma subtypes, tumor microenvironment components, and immune modulators, which will be critical for maximizing the clinical benefit of AR targeting.

Overall, AR represents a compelling and underexplored axis in melanoma pathogenesis and therapy. Leveraging this pathway might enhance current treatment paradigms and improve outcomes for patients with advanced and resistant disease.

## Figures and Tables

**Table 1:** Key Mechanisms and Implications.

Mechanism	Implication
Metastasis Promotion	AR enhances melanoma cell invasiveness by disrupting cell adhesion (e.g., FUT4-mediated junctions) and degrading MITF, a key lineage transcription factor.
Immunosuppression	AR signaling promotes immune evasion by facilitating MICA shedding and upregulating immune checkpoint molecules.
Therapy Resistance	AR activity contributes to resistance against chemotherapy, targeted therapies, and immunotherapy. AR blockade has been shown to restore sensitivity to treatment.
TME Reprogramming	AR shapes the TME into a tolerogenic state by: (i) decreasing infiltration of cytotoxic T and B cells, (ii) promoting M2 macrophage polarization and enrichment of MDSCs/Tregs, (iii) fostering exhausted CD8^+^ T cells, (iv) inducing immunosuppressive cytokines and chemokines.
Sex-Specific Differences	Sex hormones and AR signaling underlie gender disparities in melanoma incidence, progression, and treatment response. Male patients exhibit higher AR activity, which may contribute to worse outcomes compared with females.

Abbreviations: AR, Androgen Receptor; MITF, Microphthalmia-associated transcription factor; MDSCs, Myeloid-derived suppressor cells; Tregs, Regulatory T cells; TME, Tumor Microenvironment.

## Data Availability

No new data were created or analyzed in this study. Data sharing is not applicable to this article.

## Abbreviations

The following abbreviations are used in this manuscript:

AR	Androgen receptor
ICIs	Immune Checkpoint Inhibitors
UV	Ultraviolet
EMT	Epithelial-to-mesenchymal transition
TWIST1	Twist-related protein
SNAI1	Zinc finger protein SNAI1
MMPs	Matrix Metalloproteinases
ICAM1	Intracellular adhesion molecule 1
IL-6	Interleukin 6
FUT4	Fucosyltransferase 4
AJs	Adherence junctions
TCGA SKCM	The Cancer Genome Atlas human skin cutaneous melanoma
TME	Tumor Microenvironment
MICA	Major histocompatibility complex class I chain-related protein A
MICB	Major histocompatibility complex class I chain-related protein B
MHC	Major histocompatibility complex
TAP	Transporter associated with antigen processing protein
Tregs	T regulatory cells
MDSCs	Myeloid-derived suppressor cells
ADT	Androgen deprivation therapy
SARDs	Selective AR degraders
PROTACs	Proteolysis-targeting chimeras
RNAi	RNA interface
SARMs	Selective Androgen Receptor Modulators
